# Dental expenditure and catastrophic dental expenditure in Eastern Saudi Arabia: Pattern and associated factors

**DOI:** 10.4317/jced.55820

**Published:** 2019-07-01

**Authors:** Ali AlBaty, Hassan AlGhasham, Mahdi Al Wusaybie, Maha El Tantawi

**Affiliations:** 1 BDS, General Dentist. Department of Preventive Dental Sciences, College of Dentistry, Imam Abdulrahman Bin Faisal University, Dammam, Saudi Arabia; 2 BDS, MSc, PhD, Professor. Department of Preventive Dental Sciences, College of Dentistry, Imam Abdulrahman Bin Faisal University, Dammam, Saudi Arabia

## Abstract

**Background:**

Dental services have one of the highest expenses among health services. The aims of the study were to assess (1) dental expenditure (DE), (2) catastrophic dental expenditure (CDE), (3) dental services payment and (4) factors associated with DE and CDE.

**Material and Methods:**

A cross sectional study was conducted in 2018 in Saudi Arabia. Using convenience sample, participants were recruited from governmental and private dental clinics/hospitals. A questionnaire assessed (a) personal information, (b) dental background: payment methods, type of clinics visited, perceived oral health status, frequency of pain and (c) payment for dental services received. The number of remaining teeth was clinically assessed. Two outcome variables were assessed (1) total DE in linear regression and (b) CDE (DE exceeds 10% of income) in logistic regression. Personal and dental background variables were explanatory variables.

**Results:**

The response rate was 83.8% (419/500) with 43% reporting expenditure, 16.5% facing CDE and 36.3% using multiple payment methods. The greatest DE was for crowns and bridges, root canal therapy, fillings and implants. Income, payment method and pain were associated with DE and CDE.

**Conclusions:**

Participants used multiple payment methods including out of pocket and faced CDE. The bulk of expenditure was for rehabilitative services. The availability and quality of health-insured primary care services may reduce the financial burden facing dental patients.

** Key words:**Health expenditure, Saudi Arabia, dental care, insurance, dental, universal health insurance.

## Introduction

Health expenditure is money spent by individuals, groups, nations, or organizations for health care, may be equivalent to actual cost and may be shared among patients, insurers, and employers (1). Studies showed that payment methods for health care- such as insurance and out of pocket (OOP)- affect access to care, equitable distribution of resources and achievement of sustainable development goals (2). Increased health care spending was also associated with lower disease prevalence and incidence (3).

Dental expenditure (DE) has increased with greater focus on ensuring that it meets treatment needs and improves oral health (4). High cost of dental services was associated with less dental visits and deferring recommended treatment (5). This risk was higher among the poor (6) who may sacrifice other spending such as for food (7). Alternatively, covering dental care cost by insurance was associated with higher likelihood of dental visits (8). OOP spending on dental care increased due to higher treatment cost (9,10) especially in developing countries (10,11) and because of greater demand for dental care in private clinics. OOP payment may be associated with catastrophic dental expenditure (CDE) if payment for dental services exceeds 10%-20% of household income (12). This emphasizes the importance of universal health coverage (UHC) where people receive health services without exposure to undue financial burden (13).

Saudi Arabia, a high income country, has a generously funded health care system with total expenditure on health per capita= 1,194.1 US $ representing 4.7% of GDP in 2015 (14,15) compared to global average= 822.2 US $ representing 6.2% of GDP (16,17). Saudi Arabia has UHC with UHC service coverage index= 68 (18) compared to 80 in the US and western European countries (19). Little is known about how UHC impacts DE in Saudi Arabia. Identifying the pattern of DE and associated factors helps in planning health care services and provides evidence for the impact of different payment mechanisms on care provision.

The hypothesis of this study was that dental services are provided in governmental clinics, that governmental insurance pays for dental care, and that CDE is lower than in other countries without UHC. The study assessed the (1) pattern of DE, (2) prevalence of CDE, (3) payment methods for dental services, and (4) factors associated with DE and CDE.

## Material and Methods

A cross-sectional study was conducted in Saudi Arabia, Eastern Province, February to July 2018 after obtaining approval from the Research Unit, College of Dentistry, Imam Abdulrahman Bin Faisal University (#2018011). Dental patients were recruited from major cities; Dammam, AlHassa, Khobar, Qatif and Dhahran by inviting visitors of governmental hospitals (n= 4), university hospitals (n=2) and major private clinics (n=4). Participants were conveniently selected and included if they (1) were adults (>18 years old), (2) lived in Saudi Arabia during the last year, and (3) consented to participate. Sample size was estimated based on assumptions: 5% error margin, 95% confidence level, DE prevalence= 50%. The calculated (https://select-statistics.co.uk/calculators/sample-size-calculator-population-proportion/) sample size= 383.

A standardized, interview-based questionnaire based on a previous study (20) was translated to Arabic and assessed for face and content validity by experts not involved with the study. It was pilot-tested on 15 participants who were afterwards excluded from the study. Their comments were used to produce the final questionnaire. The questionnaire included several sections (a) personal information (gender (male or female), age (<40 yrs or 40+ yrs old), nationality (Saudi or non-Saudi), employment (governmental, private, self-employed, unemployed or retired) and monthly household income (up to poverty line (4,000 SAR); up to sufficiency line (8,000 SAR), up to salary of a middle ranking employee (20,000 SAR), up to salary of a high ranking employee (40,000 SAR) and more, based on King Khalid Foundation report about poverty in Saudi Arabia (21), (b) dental background (payment method for dental care (governmental insurance, private insurance and OOP), dental clinic (governmental or private), perceived oral health (4-points scale: excellent to poor) and dental pain frequency (4-points scale: always to never)). The last section (c) asked participants whether they obtained 12 dental services last year, if they paid using governmental insurance, private insurance or OOP and how much they paid in SAR (1SAR= 0.27 US$). One examiner clinically counted the number of remaining teeth under natural daylight using disposable mirrors. Root fragments were included and third molars were excluded (22).

There were two outcome variables: total DE, calculated by adding DE for all services. We also calculated mean DE per service for all participants and for those who spent money to obtain that service. We counted the number of participants receiving each service and the number by payment method. We calculated the mid-point of household monthly income categories and used these as denominators to calculate the percentage of DE to monthly household income and identified participants with the second outcome variable, (b) CDE: OOP ≥10% of household monthly income (12). We also calculated the percentage of participants with CDE at 20% and 40% (10). The number of teeth was dichotomized into 0-19, non-functional dentition and ≥20 functional dentition (23).

We developed two multivariable models (1) linear regression: the outcome variable was total DE and (2) logistic regression: the outcome variable was having CDE at 10%. The explanatory variables were those that had significant associations with the outcome variables in bivariate analysis using Mann Whitney U test and chi square/ Fisher exact tests. These variables included enabling factors (monthly household income, payment method, dental clinic) and a factor indicating the need for care (pain frequency). Regression estimates, odds ratios and confidence intervals were calculated. Significance level was set at 5%. Analysis was conducted using SPSS version 22.0 (IBM Corp., Armonk, N.Y., USA).

## Results

The questionnaire was distributed to 500 and 419 responded (response rate= 83.8%).  Table 1,  Table 1 continue shows that 89.5% were males, 54.7% <40 years, 90.5% Saudis, 47.6% government-employed, 63.7% with monthly household income >the sufficiency line (8,000SAR). The greatest portion used multiple payment methods for dental services (36.3%), 52.4% visited private clinics, 44.7% thought their oral health was good, 41.7% did not suffer dental pain last year and 91.1% had functional dentition.

**Table 1:**
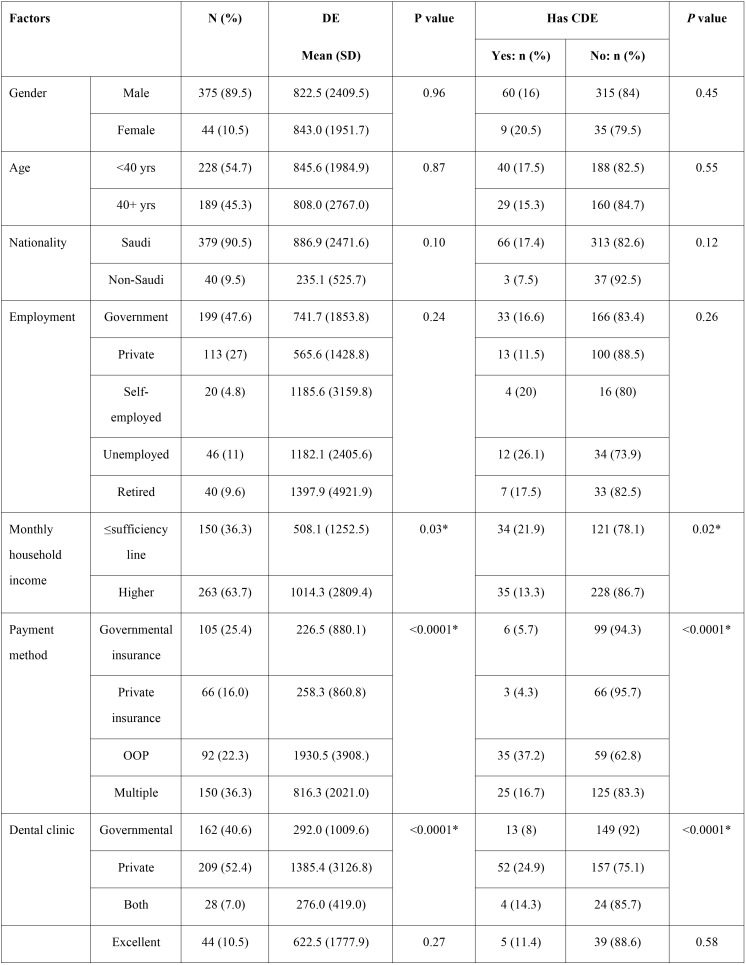
Personal, dental and expenditure characteristics.

**Table 1 continue:**
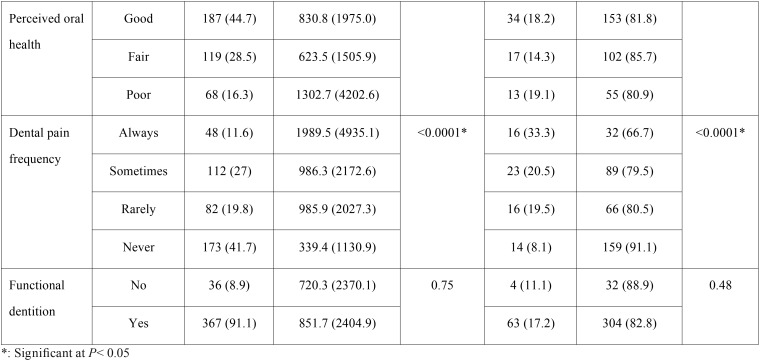
Personal, dental and expenditure characteristics.

**Figure 1:**
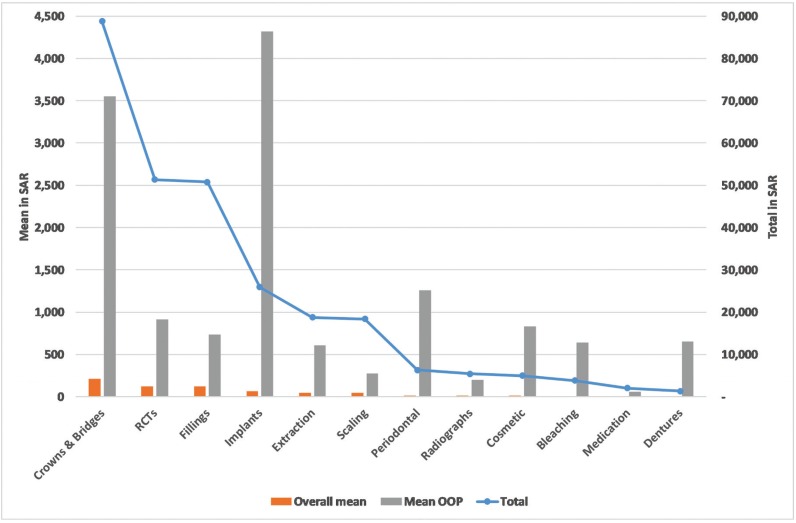
DE for service (total, overall mean and OOP).

Of all respondents, 43% had DE last year. Total DE was 345,609 SAR, overall mean (SD)= 824.8 (2363.5) SAR and median (interquartile range, IQR)= 0 (0, 450) SAR. Among those with DE, the mean (SD) = 1,920.5 (3,306.1) SAR and the median (IQR)= 705 (200,2000) SAR. Figure 1 shows that crowns and bridges had the greatest total DE (88,780 SAR) followed by root canal therapy (RCT) (51,349 SAR), fillings (50,745 SAR) and implants (25,900 SAR). The expenditure for these four services= 62.7% of total DE. DE per person ranged from overall mean= 211.9 SAR for crowns and bridges to 3.1 SAR for dentures and among those who spent money to obtain services from 1260 SAR for periodontal therapy to 61.3 SAR for medication. CDE at 10% occurred in 69 (16.5%) of participants while 10.7% faced CDE at 20% and 5.7% faced CDE at 40%.

**Figure 2:**
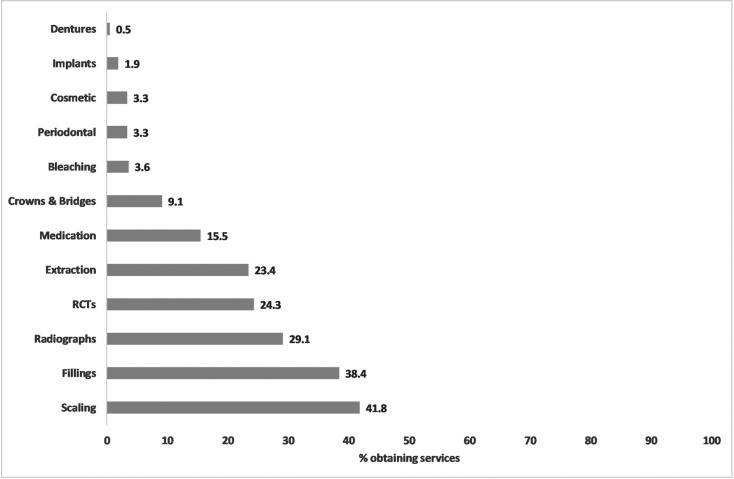
Percentage of respondents obtaining services.

**Figure 3:**
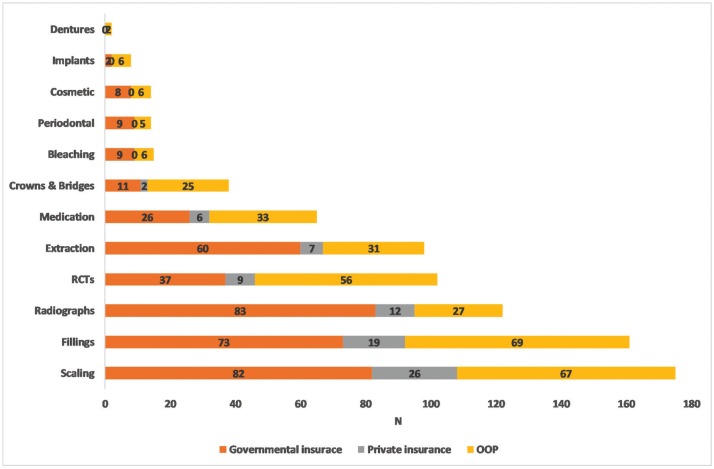
Methods of paying for services.

Figure 2 shows that last year, 41.8% obtained scaling, 38.4% fillings, 29.1% radiographs and 24.3% RCT. Figure 3 shows that governmental insurance was the main method to pay for radiographs (83/ 122, 68%), extraction (60/ 98, 61.2%) and scaling (82/ 175, 46.9%). OOP payment was used for implants (6/8, 75%), RCT (56/ 102, 54.9%), medication (33/ 65, 50.8%) and dentures (two participants). Private insurance was used to pay only for 7/ 12 services.

 Table 1 shows that respondents with monthly household income> sufficiency line (8,000 SAR) had significantly higher DE than those living below the sufficiency line (mean= 1014.3 and 508.1 SAR, P= 0.03) and significantly lower percentage of CDE (13.3% versus 21.9%, *P*= 0.02). Respondents reporting OOP payment had greater expenditure than those reporting using multiple payment methods, and governmental or private insurance (mean= 1930.5, 816.3, 226.5 and 258.3 SAR, *P*< 0.0001) and higher percentage of CDE (37.2%, 16.7%, 5.7% and 4.3%, *P*< 0.0001). Respondents who visited private clinics had higher DE than those visiting governmental clinics or both types of clinic (mean= 1385.4, 292.0 and 276.0 SAR, *P*< 0.0001) and higher percentage of CDE (24.9%, 8% and 14.3%, *P*< 0.0001). Respondents with more frequent pain had the highest DE (mean= 1989.5 SAR for those always suffering pain compared to 339.4 SAR for those who never had pain, *P*< 0.0001) and higher percentage of CDE (33.3% and 8.1%, *P*< 0.0001).

 Table 2 shows that DE was significantly higher for participants who paid OOP (B= 966.8, *P*= 0.002), those who visited private clinics (B= 1194.9, *P*= 0.01), always had pain (B= 1864.4, *P*< 0.0001), sometimes had pain (B= 702.1, *P*= 0.01) and significantly lower for participants whose monthly income was <sufficiency line (B= -582.2) and those who had private insurance (B= -926.2). Higher odds of CDE were significantly associated with having income <sufficiency line (OR= 2.35, *P*= 0.008), OOP payment (OR= 3.56, *P*< 0.0001) and feeling pain always, sometimes or rarely (OR= 6.48, *P*< 0.0001, OR= 3.11, *P*= 0.005 and OR= 3.14, *P*= 0.01).

**Table 2:**
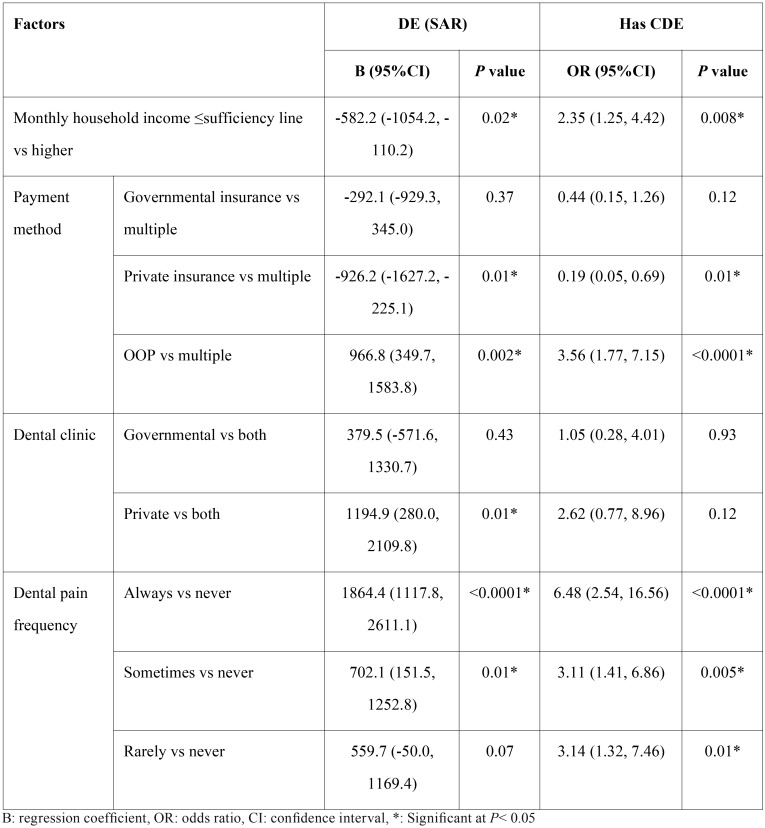
Factors associated with DE and CDE.

## Discussion

Our study showed that governmental insurance was the most frequently used single payment method for dental services although the greatest portion of participants used multiple payment methods and one out of five paid OOP for dental services. More respondents obtained care from private than governmental clinics. One out of 6 individuals faced CDE. Thus, our findings do not support our hypothesis.

The present study showed that participants used multiple payment methods for dental services; governmental insurance and OOP were equally used and more frequent than private insurance which had a minor role in financing dental services. Our finding disagrees with reports about payment methods in Canada where 31.9% of payment was OOP and 62.6% was through employment-based insurance with only 5.5% (mostly welfare recipients) eligible for public funding through government assistance programs (5). It also disagrees with an Australian study reporting that 83% of dental fees were paid by individuals (24) and a Chinese study where 80% paid OOP despite governmental and private insurance covering dental services (12).

Our results showed differences in payment method by service type. Maintenance and examination were covered by governmental insurance and formed a minor portion of DE. Rehabilitative services accounted for the bulk of DE and OOP paid for them. The reason that governmental insurance did not pay for these costly rehabilitative services may be because they were not part of the package. However, under UHC in Saudi Arabia, all services are provided to those entitled without fees (25). Another reason may be quality or availability issues preventing service use despite coverage. Previous studies showed differences in types of services by insurance status. For example, people with insurance had less extractions (26), more preventive care and more restorative services than the uninsured (27). Differences were also reported by type of insurance where patients with private insurance sought preventive services such as cleanings and check-ups (8).

Despite UHC and generous governmental health expenditure, 16.5% faced CDE at 10%. This is much higher than the level reported in a Chinese study (1.4%) (12) although the authors of that study reported that only few dental services were covered by insurance. In the present study, 5.5% faced CDE at 40%. This agrees with Masood *et al.* (10) who reported that 6.8% in Ukraine faced CDE. However, Ukraine is a middle income country whose per capita total expenditure on health is half that of Saudi Arabia’s (28). The percentage of CDE at 40% in the present study was much higher than that in India (0.6%) and Pakistan (0.5%); both low income countries, China (0.3%); a lower middle-income country and Malaysia (0.4%); an upper middle-income country. This may be explained by the observation that CDE is more common in economically-developed countries (10). Our study also disagrees with a report (29) showing major reduction in dental care cost and a decrease in CDE after implementing UHC in Thailand,.

Our results agreed with previous research showing higher DE among the wealthy (30) and higher risk of CDE among the poor (31). It also agrees with findings showing the impact of insurance on DE (24), the association of OOP with CDE (32) and with facing impoverishment (33). The association between pain and greater DE and higher odds of CDE provides evidence to advise patients about addressing dental problems at an earlier stage before they progress to pain and require complex treatment with higher cost.

Our study is limited by the convenience sample which does not allow direct generalization at a national level. Our findings may, however, be generalized to similar groups: young males, with good perception of oral health, who rarely/ never had dental pain last year and who have a functional dentition. Older people with worse oral health and women may have higher levels of DE; partly because more of them would seek dental care and partly due to more costly services to replace loss of function. The participants in the present study are similar to people seeking dental services in different facilities in Saudi Arabia. Thus, our study provides insight about dental services obtained in the country, how they are paid for and how much. Another limitation is the use of self-reporting to obtain information about service type and DE with risk of recall bias.

The present study provides evidence for health care planning and workforce training. It is important to prioritize resources and make decisions about targeting the greatest number of people who demand non-costly services or the few who demand expensive services. In these cases, concerned stakeholders from the academic sector, private and governmental service providers and patients’ representatives need to be involved in the discussion.

 Prosthetic services including crowns and bridges, implants and dentures are used to replace missing teeth. The first two services were more frequently used in the present study than the latter service although they were more expensive. Some of these differences may be attributed to clinical indications and dentists’ preferences. Another part of the difference is attributed to patients’ choices and these should be considered during treatment planning, policy setting and workforce training. Ensuring the availability and insurance coverage of primary care services (examination, scaling, extraction and simple filings) would reduce the need for costly prosthetic services. Such services may be included in private insurance packages to reduce health care system cost.

## Conclusions

Less than half the participants seeking dental care in the Eastern Province, Saudi Arabia reported DE last year and 16.5% faced CDE because they used >10% of their income to pay OOP for DE. Rehabilitative services such as crowns and bridges, RCT, fillings and implants represented 60% of DE. Most participants obtained care in private clinics using multiple payment methods. DE was higher among richer people, those paying OOP, those visiting private clinics and those with frequent dental pain. CDE was associated with poverty, paying OOP and dental pain. Ensuring the availability and insurance-coverage of basic dental services in governmental clinics may reduce the financial burden for patients in need of care.

## References

[1] MeSH: Health Expenditures. National Library of Medicine.

[2] Bose M, Dutta A (2018). Health financing strategies to reduce out-of-pocket burden in India: a comparative study of three states. BMC Health Serv Res.

[3] Dieleman JL, Squires E, Bui AL, Campbell M, Chapin A, Hamavid H (2017). Factors Associated With Increases in US Health Care Spending, 1996-2013. J Am Med Assoc.

[4] Ramraj C, Weitzner E, Figueiredo R, Quiñonez C (2014). A macroeconomic review of dentistry in Canada in the 2000s. J Can Dent Assoc.

[5] Locker D, Maggirias J, Quiñonez C (2011). Income, dental insurance coverage, and financial barriers to dental care among Canadian adults. J Public Health Dent.

[6] Pradeep Y, Chakravarty KK, Simhadri K, Ghenam A, Naidu GM, Vundavalli S (2016). Gaps in need, demand, and effective demand for dental care utilization among residents of Krishna district, Andhra Pradesh, India. J Int Soc Prev Community Dent.

[7] Thompson B, Cooney P, Lawrence H, Ravaghi V, Quiñonez C (2014). Cost as a barrier to accessing dental care: findings from a Canadian population-based study. J Public Health Dent.

[8] Cooper PF, Manski RJ, Pepper J V (2012). The Effect of Dental Insurance on Dental Care Use and Selection Bias. Med Care.

[9] Petersen PE, Bourgeois D, Ogawa H, Estupinan-Day S, Ndiaye C (2005). The global burden of oral diseases and risks to oral health. Bull World Health Organ.

[10] Masood M, Sheiham A, Bernabé E (2015). Household Expenditure for Dental Care in Low and Middle Income Countries. Milgrom PM, editor. PLoS One.

[11] McIntyre D, Thiede M, Dahlgren G, Whitehead M (2006). What are the economic consequences for households of illness and of paying for health care in low- and middle-income country contexts?. Soc Sci Med.

[12] Sun X, Bernabé E, Liu X, Gallagher JE, Zheng S (2016). Determinants of Catastrophic Dental Health Expenditure in China. PLoS One.

[13] (2016). Health systems financing: the path to universal coverage. WHO.

[14] (2019). Saudi Arabia. WHO.

[15] Current health expenditure (CHE) per capita in US$ - Data by country. WHO.

[16] Current health expenditure (CHE) per capita in US$ - Data by WHO region. WHO.

[17] Current health expenditure (CHE) as percentage of gross domestic product (GDP) (%) - Data by WHO region. WHO.

[18] Global Health Observatory. WHO.

[19] Global Health Observatory. WHO.

[20] Ramraj C, Quiñonez C (2013). Self-reported cost-prohibitive dental care needs among Canadians. Int J Dent Hyg.

[21] Determining poverty line and sufficiency line Developing the Government Subsidy System in the Kingdom of Saudi Arabia. http://www.kkf.org.sa/en/Documents/PovertyReportFinal-EN.pdf.

[22] (2010). Association of State and Territorial Dental Directors. Basic Screening Surveys: An approach to monitoring community oral health. http://www.prevmed.org/wp-content/uploads/2013/11/BSS-SeniorsManual.pdf.

[23] (2013). Shortened dental arch therapy in old age. https://bda.org/dentists/education/sgh/Documents/Shorteneddentalarchtherapyinoldage.pdf.

[24] Teusner D, Brennan D, Gnanamanickam E (2013). Individual dental expenditure by Australian adults. Aust Dent J.

[25] Almalki M, Fitzgerald G, Clark M (2011). Health care system in Saudi Arabia: an overview. East Mediterr Health J.

[26] Bayat F, Murtomaa H, Vehkalahti MM, Tala H (2011). Does dental insurance make a difference in type of service received by Iranian dentate adults?. Eur J Dent.

[27] Meyerhoefer CD, Zuvekas SH, Manski R (2014). The demand for preventive and restorative dental services. Health Econ.

[28] (2019). Ukraine. WHO.

[29] Yiengprugsawan V, Kelly M, Seubsman S A, Sleigh AC (2010). The first 10 years of the Universal Coverage Scheme in Thailand: review of its impact on health inequalities and lessons learnt for middle-income countries. Australas Epidemiol.

[30] Pérez-Núñez R, Vargas-Palacios A, Ochoa-Moreno I, Medina-Solis CE (2007). Household expenditure in dental health care: national estimations in Mexico for 2000, 2002, and 2004. J Public Health Dent.

[31] Proaño Falconi D, Bernabé E (2018). Determinants of catastrophic healthcare expenditure in Peru. Int J Heal Econ Manag.

[32] Kavosi Z, Rashidian A, Pourreza A, Majdzadeh R, Pourmalek F, Hosseinpour AR (2012). Inequality in household catastrophic health care expenditure in a low-income society of Iran. Health Policy Plan.

[33] Bernabé E, Masood M, Vujicic M (2017). The impact of out-of-pocket payments for dental care on household finances in low and middle income countries. BMC Public Health.

